# Antibiotic Resistance Profile of *E. coli* Isolates from Lettuce, Poultry Manure, Irrigation Water, and Soil in Kumasi, Ghana

**DOI:** 10.1155/2024/6681311

**Published:** 2024-01-27

**Authors:** Abigail Abena Anokyewaa Appau, Linda Aurelia Ofori

**Affiliations:** Department of Theoretical and Applied Biology, Kwame Nkrumah University of Science Technology, Kumasi, Ghana

## Abstract

Inputs such as irrigation water and poultry manure used in lettuce cultivation have been found to be associated with antibiotic-resistant pathogens. The study assessed the antibiotic resistance profile of *Escherichia coli* isolated from lettuce, poultry manure, irrigation water, and soil in Kumasi. One hundred and fifty-six samples of lettuce, irrigation water, soil, and manure were collected from three farms over a seven-week cultivation period (seedlings to harvest stage). *E. coli* were enumerated using standard methods. 98% of the samples were positive for *E. coli*. Geometric means for lettuce, irrigation water, and soil ranged from 2.0 × 10^5^ to 1.67 × 10^7^ MPN/100 ml while that of manure ranged from 2.0 × 10^5^ to 1.31 × 10^7^ MPN/100 ml. Generally, the microbial load of all parameters on all farms across the weeks was significant and exceeded World Health Organization (WHO) and International Commission on Microbiological Specifications for Foods (ICMSF) standard recommendations for food. Using the Kirby Bauer method, antibiotic sensitivity testing was performed against 225 biochemically confirmed *E. coli* with twelve antibiotics. Relatively high resistance was recorded for some members of the beta-lactam class: meropenem: 94.2%, ampicillin: 91.9%, cefuroxime: 95.1%, ceftriaxone: 94.7%, and cefotaxime: 94.2%. Eighty of the isolates were screened for extended spectrum beta lactamase (ESBL) production using cefotaxime (CTX) and cefotaxime/clavulanic acid (CTX/CLA) discs and three showed positive: one each from poultry manure, irrigation water, and soil. Polymerase chain reaction (PCR) confirmed the presence of bla_CTX-M_ gene. The occurrence of antibiotic-resistant *E. coli* in vegetables and their production environment is alarming and poses serious health threats to the general public. The presence of bla_CTX-M_ gene in *E. coli* from a vegetable production site recorded for the first time in Ghana requires enforcement by regulatory bodies on the inappropriate use of antibiotics in the country.

## 1. Introduction

Antimicrobial resistance (AMR) is the potential of a microbe (usually a bacteria) to resist the effects of a medicine that was previously used to treat that microbe. Disease-causing organisms: pathogens naturally develop resistance but the abuse of antimicrobial substances increases the frequency of the emergence of AMR and further spread resistance genes in the microbes. With regards to this, medicines that were previously used to treat infections effectively become less effective or totally ineffective [1].

This results in prolonged infections and increased mortality as well as production loses. An outbreak of a multiresistant strain *E. coli* 0157: H7 in 1991 caused hospitalization of 71 people out of whom 2 died in New York [2]. Similarly, in Canada, outbreaks of *E. coli* 0157: H7 strain and *Campylobacter jejuni* in drinking water from contaminated underground water sources also resulted in 2750 cases, leaving 7 out of the 65 hospitalized dead [3]. According to the Food and Agriculture Organization of the United Nations [1], about 700 thousand people die each year as a result of antimicrobial resistance and, if the issue of AMR is not solved, this could increase to 10 million in the coming years.

Currently, AMR is a major global threat to both human and animal health and it is influenced by the excessive and inappropriate use of antibiotics [4]. Antibiotics have been used not only in human medicine but also in animal production (especially poultry farming) since the introduction of penicillin in the early twentieth century. Formerly, it was used for therapeutic purposes (prophylaxis) to treat sick animals and later as feed additives to promote growth at low concentrations [1, 5]. This will promote the development of resistant types of microorganisms by selection pressure [6]. The increasing human population and the high demand for animal products and eggs across the globe will escalate the use of antibiotics.

About 90 percent of these antibiotics may be excreted unmetabolized in water and animal waste and can thereafter spread into the environment. Subsequently, microbes associated with poultry may develop AMR genes through constant exposure to antimicrobial residues and further spread of resistance through transfer of resistance genes to other microorganisms [5].

The frequent use of antibiotics in poultry is likely to favor some pathogenic bacteria in the poultry (chicken) or in soils where antibiotics-contaminated poultry manure is being applied. Studies by Kumar et al. [7] and Van and Blaney [8] recorded some classes of commonly used antibiotics excreted in poultry manure. Consumption of antibiotics-contaminated foods will increase antibiotic resistance, which may further lead to food poisoning or allergies. According to Danilova et al. [9], chicken manure contributes highly to antibiotic-resistant organisms in soil compared to other livestock. There has been an increased use of poultry manure in vegetable production.

An effective and cheap way of disposing waste accumulated in the poultry industry is manure. In Ashanti region and most periurban areas in Ghana, poultry manure is used due to its high levels of nitrogen and other trace elements in its composition. Nevertheless, studies by [10] revealed that poultry manure is also contaminated with pathogenic microorganisms which may or may not confer antimicrobial resistance and residues of antibiotics.

In urban areas such as Accra and Kumasi, most vegetable farmers resort to wastewater for irrigation. The waste water could comprise of urine, feces, wastewater from kitchens, laundries, leaking septic tanks, and industrial waste [11]. Helminth infections, diarrhea, and other foodborne diseases have been associated with wastewater use for irrigation although its disease transmission is unclear [12]. Previously, possible antibiotic residues have also been found in irrigation water used in Kumasi for vegetable farming [13].

According to Amponsah-Doku et al. [14], vegetables grown in urban areas such as lettuce are mostly contaminated with highly pathogenic microorganisms which are multidrug resistant. This poses serious health threats to the consumer and affects food security at large. Attaining information on the profile of antibiotic resistance of the organisms associated will aid public health interventions.

This study aimed at assessing the microbial load of *E. coli* on lettuce, irrigation water, poultry manure, and soil across the lettuce growth period and also assessed the resistance profile of isolates against some selected antibiotics.

## 2. Materials and Methods

### 2.1. Study Area and Sample Size

The study involved a longitudinal research design carried out from November 2020 to August 2021. Antibiotic resistance profiles of *E. coli* on three lettuce farms at Kotei-Deduako, Boadi, and KNUST, all located in Kumasi, Ghana, were studied. The study areas have coordinates 6°40′N 1°37′W. Kumasi is one of the largest cities in Ghana with a population of 5,400,000 [15]. The average temperature of the three study sites is about 26°C with an average rainfall of 1314.7 mm and relative humidity (RH) of 73%.

Under favorable conditions, lettuce takes an average of 6 to 8 weeks to mature and be ready for harvest. In this study, the average time for which all lettuce took to mature was 7 weeks. In the first two weeks of the growth process, the lettuce seedlings remain on a seedling bed. During this period, there is no manure application since the manure has high ammonia and can cause the seedlings to die. After the seedling stage, the seedlings are transplanted onto larger beds and spaced out to make room for growth. Manure is applied from the 3^rd^ week upwards to support plant growth. This sampling procedure was employed to study the microbial load of the samples across the lettuce growth period.

Forty-two samples of lettuce, irrigation water, soil, and 30 samples of poultry manure were collected. For the purpose of replication, two samples of lettuce, soil, and water were collected from week 1 to week 7 for all three farms, whereas 2 samples of manure were collected from week 3 to week 7 from all three farms. This is because manure was only applied after the seedlings were transplanted to the main planting beds (week 2).

No ethical clearance was sought for this research because no human or animal participants were used.

### 2.2. Sample Collection

Prior to sample collection, farmers gave their consent to use their farms for the study. On sample collection day, two samples of lettuce, poultry manure, irrigation water, and soil were collected from all three farms. All farms had an average size of about 80 km × 40 km. Lettuce, manure, and soil samples were picked with sterile gloves and were put into well–labelled sterile ziplock bags. Lettuce samples were selected randomly from the designated bed (the particular batch of lettuce which were being studied over the 7-week period), whereas soil samples (loamy soil) with a depth of 2 cm on the same bed were collected. About 20 grams of poultry manure which was ready to be applied on the beds were also taken for analysis. Water samples were collected with sterile plastic bottles by dipping it some few centimeters (2–5 centimeters) below the water surface to avoid collecting debris on the surface. To prevent contamination, all samples were picked with different gloves. All samples were preserved in their raw state and transported on ice in a cool box within two hours to the laboratory for analysis.

### 2.3. Isolation and Identification of *E. coli*

10 g of lettuce, soil, and manure were weighed into well-labelled sterile zip lock bags and 90 ml of peptone water (Oxoid, UK) was added and pulsified using a pulsifier (PUL 100E; Stuart Scientific Co. Ltd., UK) for 30 s. One millimeter (1 ml) of the resultant stock solution was inoculated in 5 ml of MacConkey broth (Oxoid, UK) and incubated for 24 ± 2 hours at 44°C. 1 ml of the overnight positive culture was transferred into test tubes containing 5 ml of Tryptone Soy Broth (Oxoid, UK) and incubated for 24± 2 hours at 44°C. A few drops of Kovac's Indole Reagent (LOBA CHEMIE PVT. Ltd., India) were added to each positive tube. All tubes that retained the red color ring following simple agitation were selected, and a loop full streaked on Eosin Methylene Blue Agar (EMBA) (Oxoid, UK) and incubated for 24± 2 hours at 37°C. Colonies with a shiny green metallic appearance were confirmed as *E. coli* and stored in Brain Heart Infusion (Oxoid, UK) with 20% glycerol.

### 2.4. Antibiotic Sensitivity Testing of *E. coli*

The Kirby Bauer disc diffusion method was used with 12 antibiotic discs from Biomark Laboratories (ISO certification: ISO 9001-2015 and ISO 13485-2016) while following the Clinical and Laboratory Standard Institute guidelines (“M100-S11, Performance Standards for Antimicrobial Susceptibility Testing,” 2019). The antibiotics tested were AMP: ampicillin-10 *μ*g, TET: tetracycline-10 *μ*g, COT: cotrimoxazole-25 *μ*g, CRX: cefuroxime-30 *μ*g, VAN: vancomycin-30 *μ*g, CHL: chloramphenicol-10 *μ*g, CTR: ceftriaxone-30 *μ*g, CTX: cefotaxime 30 *μ*g, CIP: ciprofloxacin-5 *μ*g, and MEM: meropenem-10 *μ*g. Isolates were thawed from the freezer and streaked on Muller–Hinton agar for incubation at 37°C for 24 hours. Using a sterile swap, 2 to 3 distinct colonies were picked to make a suspension at 0.5 McFarland in normal saline. This was spread on Muller–Hinton agar, and the disc placed on it with the aid of forceps. The samples were incubated at 37°C for 18 to 24 hours. Zone diameters of each of the antibiotic discs were measured, and the results were interpreted with the CLSI breakpoints.

### 2.5. ESBL Screening

*E. coli* isolates were screened for ESBL using a combination disc test (CDT) with cefotaxime in combination with clavulanic acid and interpreted using CLSI standards recommendations. A 0.5 McFarland standard of the isolates was prepared using fresh pure cultures. Samples were spread on a plate containing Muller–Hinton agar (Oxoid, UK) with the aid of a sterile cotton swab. The antibiotic discs: CTX: cefotaxime (30 *μ*g) and CTX/CLA: cefotaxime/clavulanic acid (10 *μ*g) (all Oxoid, UK), were placed on the plate while ensuring that there were enough spaces between the individual discs to enhance accurate measurements of the zones of inhibition. The plates were incubated at 35 ± 2 °C for 18 to 24 hours.

### 2.6. DNA Extraction

DNA was extracted using the boiling method. *E. coli* isolates were cultured on blood agar and a single colony grown on nutrient agar (Oxoid, UK). Two distinct colonies were placed in an Eppendorf tube containing 1 ml of phosphate-buffered saline (PBS) (Sigma Aldrich Inc., USA), vortexed and centrifuged at 8000 rpm for 3 min. The supernatant was discarded after which 100 *μ*L of nuclease-free water was added. The mixture was vortex again and boiled (using a heating block at 95°C) for 5 minutes. It was later centrifuged at 14000 rpm for 3 min. 100 µL of the supernatant, which is the bacteria DNA, was pipetted into a new Eppendorf tube and the pellet was discarded.

### 2.7. PCR Detection of bla_CTX-M_ Gene

The primer used for the detection of the bla_CTX-M_ gene was described by Bauernfeind et al. [16]. Sequences for forward and reverse reactions were 5′-GCGATGTGCAGCACCAGTAA-3′ and 5′-GGTTGAGGCTGGGTGAAGTA-3′, respectively, with an expected band size of 500 to 600 bp. The optimized PCR conditions used included an initial denaturation step at 94°C for 3 min, followed by 35 cycles of denaturation at 94°C for 45 s. Annealing was done at 62°C for 45 s, extension at 72°C for 30 s, and a final extension at 72°C for 10 minutes with 4°C for holding. The amplified products were loaded in a 1.5% agarose gel with a 100 bp molecular weight DNA gene marker as a ladder. Electrophoresis was done at 100 amp for 40 minutes. Ethidium bromide (1 *μ*g/mL) was used to stain the amplified DNA fragments for visualization under UV light illumination. Both positive (CTX-M-positive*E. coli*) and negative (nuclease-free water) controls were employed in carrying out these procedures.

### 2.8. Data Analysis

The percentages of antibiotic sensitivities were generated using Microsoft Excel whereas the geometric means, ranges, and standard deviation (Tables 1 and 2) were also analyzed with SPSS version 23. ANOVA was also used to generate associations between the weeks and parameters using R code/software.

## 3. Results

### 3.1. Microbial Analysis

The geometric means for *E. coli* load on lettuce, manure, soil, and irrigation water for all the farms ranged from 2.00 × 10^5^ to 1.67 × 10^7^ MPN/100 ml (Table 1). On the KNUST farm, soil recorded the highest microbial load of 6.57 × 10^6^ MPN/100 ml, whereas irrigation water recorded the lowest of 3.25 × 10^6^ MPN/100 ml. On the contrary, the highest microbial load was recorded for irrigation water (4.68 × 10^6^ MPN/100 ml) and the lowest (1.83 × 10^6^ MPN/100 ml) for lettuce. Lettuce, however, recorded the highest microbial load at Boadi (5.62 × 10^6^ MPN/100 ml) whereas manure recorded the lowest (2.97 × 10^6^ MPN/100 ml) (Table 1).

Total microbial load of lettuce differed significantly over the weeks. Specifically, weeks 4 and 6 as well as weeks 2 and 7 had similar microbial loads but differed significantly from the microbial load in weeks 1, 3, and 5. However, the lowest microbial contamination was recorded in week 5 (1.24 × 10^6^ MPN/100 ml), and the highest in week 3 (1.06 × 10^7^ MPN/100 ml) (Table 2).

### 3.2. Antibiotic Sensitivity

A total of 225 biochemically confirmed *E. coli* isolates (60: lettuce, 41: manure, 61:water, and 63: soil).

The highest resistance was recorded for cefuroxime (95.1%), cefotaxime (94.7%), ciprofloxacin (91.5%), and meropenem (94.2%) whereas the lowest was recorded for chloramphenicol (89.4%) (Table 3).

#### 3.2.1. Percentage Resistance of *E. coli* Isolates from Lettuce, Manure, Soil, and Irrigation Water

*E. coli* isolated from lettuce leaves showed high resistance to ceftriaxone (95%), cefuroxime (93.3%), and vancomycin (93.1%) (Table 4).

Isolates from manure also showed resistance to cefuroxime (90.3%), vancomycin (87.8%), ceftriaxone (85.4%), meropenem (85.4%), and cefotaxime (85.4%) (Table 5).

Isolates from soil also were resistant to vancomycin (98.4%), cefotaxime (98.4%), chloramphenicol (96.9%), and meropenem (98.4%) (Table 6).

The isolates showed high resistance to all antibiotics, notably cefuroxime, vancomycin, cefotaxime, and meropenem all recorded 98.4% resistance except for chloramphenicol 90.1%. Highest susceptibility was also recorded for chloramphenicol as well 9.9% (Table 7).

### 3.3. Antibiotic Sensitivity Profiles

Four percent (4%) of the *E. coli* isolates were susceptible to all 12 antibiotics used, whereas the rest (96%) were resistant to at least 7 antibiotics making them multidrug-resistant pathogens. Twenty-seven resistance patterns were derived from the 225 isolates with AMPTETCOTCRXVANCHLCTRCTXCIPMEM being the most occurring resistant pattern; 212 out of 225 *E. coli* isolates were resistant to cefotaxime which is an important factor for screening for pathogens that produce ESBL (Table 8).

### 3.4. ESBL Screening and Confirmation

**Figure 1:**
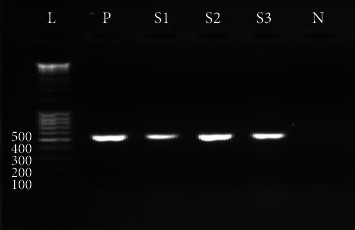
Gel electrophoresis amplification of bla_CTX-M_ genes in *E. coli* isolates (586 bp). L: DNA ladder (100 bp), P: positive control, S1: *E. coli* isolated from manure, S2: *E. coli* isolated from water, and S3: *E. coli* isolated from soil.

Three (3.75%) out of 80 isolates that were screened using CTX and CTX/CLA antibiotic disc had a difference greater than 5 mm, implying possible ESBL production. All three isolates: 1 each from manure, irrigation water, and soil expressed the bla_CTX-M_ gene after PCR. Below is a figure representing the amplified product of the bla_CTX-M_ genes (Figure 1).

## 4. Discussion

### 4.1. Microbial Analysis

Microbial contaminations occur during production processes, harvest, and even handling before they reach the consumer with chances of the presence of pathogenic microorganisms. Fresh leafy vegetables have been linked to the spread of foodborne illnesses all over the world. The presence of higher bacteria loads of *E. coli* on vegetable food items is a sign of fecal contamination and poor sanitation with higher chances of zoonotic pathogens being present [17]. As an indication of fecal contamination, the levels of *E. coli* in vegetables and their production environment are highly relevant.

The microbial loads on lettuce, irrigation water, manure, and soil in this present study confirm studies previously done by Keraita et al. [13], Hassan et al. [18], Holvoet et al. [19], Amponsah-Doku et al. [14], and Amoah et al. [12] on high loads of bacteria on lettuce and the production environment. In the current study, all parameters had microbial loads ranging from 2.0 × 10^5^ to 1.67 × 10^7^ MPN/100 ml which is approximately 5.30 to 7.22 Log MPN/100 ml (Table 1). Contrary to this study, Holvoet et al. [19] recorded relatively low *E. coli* counts in soil, irrigation water, and lettuce samples (0.7 to 3.2, 0 to 3.6, and 0.7 to 2.0 log CFU/ml, respectively). Similarly, a study by Antwi-Agyei et al. [20] recorded *E. coli* counts between 0.64 to 3.84 log CFU/g on fresh produce (lettuce) in Accra, Ghana. In Ethiopia, Weldezgina and Muleta [21] recorded counts between 5.0 and 8.36 log CFU/g of Enterobacteriaceae from irrigation water sources and fresh vegetables. Reports from Drechsel et al. [22] on fresh poultry litter used for cultivating vegetables in Kumasi, Ghana, indicated that they contained fecal coliforms between 3.6 × 10^4^ and 1.1 × 10^7^ CFU/g which is higher than *E. coli* counts recorded in the current study.

Statistically, there was a significant difference (Table 2) in microbial loads of lettuce across the seven weeks (*p* < 0.05). Weeks 1, 3, and 5 were significantly different from all other weeks (*p* < 0.05). Similarly, weeks 2 and 7 were statistically the same as weeks 4 and 6 (*p* > 0.05). Generally, microbial loads differed significantly over time (weeks). Variations in microbial loads on lettuce across the weeks could be attributed to the source of irrigation water and soil as well as manure.

Microbes can be transferred to lettuce through the vascular bundles and during irrigation via splashes [23]. Although soil is a natural habitat for many microorganisms, the loads of sources of irrigation water and manure contribute greatly to the levels of microorganisms present. On the other hand, microbial loads in manure could also be attributed to changes in storage conditions of manure at the farm and source of manure, poultry farm-specific, and the type of bedding materials used in the poultry farm [24]. Fluctuations in microbial loads in irrigation water could be attributed to variations in the composition of the irrigation water. Irrigation water could contain more sewage at one point and less at another point since it is not a stagnant water body [25].

### 4.2. Antibiotic Sensitivity

Food pathogens such as *E. coli* have developed resistance to several antibiotics which is now a global health issue. The usage of antibiotics in agricultural techniques has imparted greatly to antibiotic resistance development. Antibiotics are the basic treatments given for human and animal infections and could trigger some of the bacteria to develop resistance to the antibiotics which were used to manage them. Maka et al. [26] confirmed that single usage of an antibiotic may aid the development of antibiotic resistance to the same antibiotic class, a different class, or possibly other antimicrobial substances. These pathogens may subsequently enter water bodies and finally be used in irrigating vegetables for consumption. Vegetables eventually get contaminated with resistant pathogens and affect the whole food chain.

Globally, bacteria phenotypes with similar multidrug resistance characteristics have been observed on fresh vegetable [27–29]. The current study confirmed the presence of antibiotic-resistant pathogens on vegetables, especially lettuce grown with contaminated irrigation water or wastewater and poultry manure on contaminated soils and gives some baseline information to compare with previous studies although direct comparisons are usually hindered by different antibiotics and methods used. 9 out of the 225 isolates tested, which is approximately 4%, showed complete susceptibility to all 12 antimicrobials used. The remaining 216 (96%) isolates showed resistance to not less than 3 antibiotic classes rendering them multidrug resistant. All *E. coli* isolated from lettuce showed relatively high resistance to all 10 antibiotics which were used with AMPTETCOTCRXVANCHLCTRCTXCIPMEM being the most common resistance pattern (73.83%). The presence of antibiotic-resistant *E. coli* on lettuce samples confirms studies previously done by Österblad et al. [29] and Schwaiger et al. [30].

The study recorded extremely high resistance to some members of the beta-lactam class such as ampicillin: 91.1%, cefuroxime: 95.1%, ceftriaxone: 94.7%, cefotaxime: 94.2%, and meropenem: 94.2% which contradicts other studies. A typical example is seen in studies by Holvet et al. [19], where only 1 out of 473 isolates showed resistance to cefotaxime and 7% (including isolates from lettuce and its growing environment) to ampicillin. In Finland, Österblad et al. [29] similarly recorded no resistance for cefotaxime and imipenem. Previous studies by Schwaiger et al. [30] in Germany recorded full susceptibility to two beta-lactams: cefotaxime and imipenem. However, a recent study by Adzitey [31] in Tamale, Ghana, recorded high resistance for ampicillin: 86.67% but 40.0% resistance for ceftriaxone on ready-to-eat lettuce fields [31].

The presence of multidrug-resistant pathogens on lettuce could be due to the transmission of multidrug-resistant pathogens present in irrigation water, poultry manure, and soil during irrigation or land application of manure. Emerging pathogens resistant to penicillin have been reported decades ago and have been attributed to two enzymes: penicillinase and amidase. These enzymes hydrolyze penicillin and render its antibiotic activity negligible. However, Eriksson-Grennberg et al. [32] reported that the inoculum size used in antibiotic sensitivity tests had a significant impact on the minimum inhibitory concentration since more cells release more enzymes [32].

Resistance levels to 2^nd^ generation (cefuroxime) and 3^rd^ generation (ceftriaxone, cefotaxime) cephalosporins on both lettuce and its production environment: irrigation water, poultry manure, and soil were significantly high. This is in agreement with findings from Li et al. [33] in China and Donkor et al. [34] in Ghana on cefuroxime and cefotaxime resistance in Enterobacteriaceae isolates from livestock (food animals). However, Rasheed et al. [35] reported 8% (*n* = 99) resistance to cefotaxime from food sources in contrast to this study [35].

Resistance to carbapenems has increased over the past decade, especially in strains of Enterobacteriaceae, and has become a global health concern. Reports from Yao et al. [36] and Xu et al. [37] confirmed the presence of carbapenem-resistant *E. coli* [36, 37]. The emergence and prevalence of carbapenem-resistant *E. coli* have also been identified in some provinces in China. Similar to this study, Liang et al. [38] reported 100% resistance to imipenem, meropenem, cefotaxime, cefepime, and ampicillin in *E. coli* isolates from hospital sources: feces, urine, and sputum [38]. In India, Murugan et al. [39] recorded 23.30% resistance to meropenem from the feces of calves [39]. Ohene Larbi et al. [40] recently recorded 100% meropenem resistance in *E. coli* isolates (*n* = 43), from pig sources.

Contrary to studies by Adzitey [31], Rasheed [35], and Österblad et al. [29] who recorded low resistance to ciprofloxacin in fresh vegetables, this study recorded very high resistance to quinolones (ciprofloxacin) 91.5%. Although very few studies have been done about the movement of antibiotic residues from soil to plants, studies by Lillenberg et al. [41] revealed that 90 to 100% of ciprofloxacin was adsorbed onto lettuce [41]. Quinolone resistance is mostly facilitated by plasmid-mediated quinolone resistance, chromosomal mutations in DNA gyrase, and topoisomerase.

Contrary to Adzitey [31] who recorded 72.2% resistance to tetracycline, 90.6% was also recorded for tetracycline on *E. coli* isolates in the current study. In Lebanon, Faour-Klingbeil et al. [42] recorded 42% tetracycline resistance in *E. coli* isolates from fresh vegetables. However, *E. coli* isolates from poultry recorded resistance of 92.7%, 95.7%, and 87.9% from Adzitey [43], Larbi et al. [44], and Li et al., [33]. Studies from Sekyere [45] in Ashanti Region showed that about 60% (*n* = 108) of farms used tetracycline in pig farming. Tetracycline has been used in animal husbandry over the years for prophylaxis.

Vancomycin resistance has usually been associated with pathogens from hospital sources; however, this study recorded 95.1% which is quite significant. Studies by Gastmeier et al. [46] on vancomycin resistance development in enterococci in German hospitals reported that there were large variations in resistance to vancomycin due to situations in the four states. Cotrimoxazole as classified by the Food and Drugs Authority (FDA) of Ghana in 2015 is an over-the-counter-antibiotic. Similar to this study, Newman et al. [47] recorded 73% resistance to cotrimoxazole in Enterobacteriaceae. Adeleke and Omafuvbe [48] also recorded 60% resistance to cotrimoxazole in *E. coli* isolates from poultry feces. Donkor et al. [34] similarly recorded high resistance to cotrimoxazole in poultry.

Antibiotic resistance genes (ARGs) living on mobile genetic components or elements are found in abundance in manure. Bacteria harboring ARGs and antimicrobial substances together with their metabolites enter the soil during manure application. Upon entry into the soil, the ARGs are more likely to undergo horizontal transmission with the soil bacteria, which is a procedure facilitated by manure. The presence of antibiotic residues in food poses serious health risks due to recent reports of increased antimicrobial resistance. Food pathogens have developed various resistance patterns to antibiotics over the years. There could be occurrences of similarities or differences in the resistance patterns of isolates from the same or different food sources. Reports from Adzitey [49] show that the bacteria species involved, the type of production system employed in animal production, geographical location, sampling period, sampling technique, and the degree of antibiotic usage can greatly influence differences in antibiotic resistance profiles.

*E. coli* is one of the regular inhabitants of the gastrointestinal tract of both animals and humans [6]. While the majority of *E. coli* strains are not harmful, some can lead to severe diseases in animals, including septicemia, lung infections, gastrointestinal infections, and urinary tract infections [50]. Third-generation cephalosporins, fluoroquinones, and sulfamethoxazole are among the common antibiotics used for such infections [51]. Resistance to such antibiotics by *E. coli* is a public health issue which may have a negative influence on the country's healthcare systems and a large financial burden, prolonged hospital stays, and the necessity for more costly and intense care treatments can lower output or productivity [6]. A more alarming situation in the healthcare system is the prognosis for patients in who are immunocompromised or in critical conditions has changed due to emerging patterns of bacterial drug resistance. Doctors and other healthcare givers are now faced with more and more difficulties in giving their patients appropriate antibiotic regimens while preventing the development of additional drug resistance [52].

### 4.3. ESBL Confirmation and Genotyping

Globally, CTX-M genes have been found in *E. coli* and *Salmonella* isolates from hospital sources, humans and their excreta, animals, and animal farms [35, 53]. In most regions of Africa [50] and Ghana [54], *E. coli* isolates from livestock showing ESBL resistance commonly express the CTX-M gene. Studies in the UK recorded the gene in a water body [55] and from a chicken meat [56]. Similarly, in Iran, the CTX-M gene was identified in 79.8% of *E. coli* isolates from urban wastewater [57]. Additionally, the results of another investigation in Iran indicate that surface waters and wastewater are important reservoirs of *E. coli* with pathogenic characteristics, high levels of antibiotic resistance, and ESBL genes carriers [58].

The presence of CTX-M gene in *E. coli* from the soil at a vegetable production site has also been reported in France [59]. In the Netherlands, cases of CTX-M on *E. coli* from humans and poultry have similarly been recorded [53]. The current study for the first time in Ghana, reports the isolation presence of CTX-M gene in *E. coli* isolates from the vegetable production environment. The presence of these genes from various sources could be attributed to poultry manure and irrigation water. *E. coli* from poultry has been associated with CTX-M genes [40] and could potentially transfer them to soil or through their excreta. These genes could also come from the irrigation water since they contain hospital sewage and waste from pharmaceutical sources.

ESBL-producing bacteria have been concomitantly associated with multidrug resistance, thus conferring resistance to more than two or more classes of antibiotics [60]. The occurrence of ESBL-producing *E. coli* in vegetable production environment shows a potential transmission reservoir for livestock infections and human infections [61]. In the current study, ESBL bacteria can be transmitted directly when contaminated vegetables are ingested by humans and livestock. This will result in persistent, difficult-to-treat infections, and increase mortality.

## 5. Conclusion

The microbial loads of *E. coli* on lettuce grown in Kumasi and its production environment (irrigation water, manure, and soil) are quite alarming. All loads recorded exceed the ICMSF [62] and WHO [63] acceptable limits (10^3^) for bacterial loads on production to consumer chain. Lettuce contamination during its production period is greatly influenced by its production environment, most especially the source of irrigation water and the use of poultry manure as a nitrogen source.

The study also recorded relatively high resistance to some antibiotic classes of medical importance such as beta-lactams and quinolones. This indicates the extent to which antibiotics are being abused not only in animal husbandry but in our everyday lives. The study unequivocally highlights the gut pathogen; *E. coli* as an essential reservoir for the ESBL gene, CTX-M, which for the first time in Ghana is being isolated from a vegetable production environment.

The occurrence of antibiotic-resistant *E. coli* in vegetables and their production environment is alarming and poses serious health threats to the general public. The presence of bla_CTX-M_ gene in *E. coli* from a vegetable production site recorded for the first time in Ghana requires enforcement by regulatory bodies on the inappropriate use of antibiotics in the country. Antibiotics use in livestock should be regulated together with ensuring the use of and safe clean water for irrigation to ensure safe farming and healthy agricultural produce.

### 5.1. Limitations

Due to financial constraints, the study was unable to use highly sensitive techniques such as sequencing to study other ESBL genes such bla_SHV_ and bla_TEM_ which are of clinical significance as well as identification of plasmids which play an important role in resistance transmission. Another setback includes unwillingness of lettuce farmers to permit sample collections from their farms with the notion that, their businesses may be terminated after analysis of their produce.

## Figures and Tables

**Table 1:** Bacterial counts, geometric means, and ranges of *E. coli* isolates from lettuce, manure, soil, and irrigation water on all farms (*n* = 156).

Sampling site	Parameter	Geometric mean (MPN/100 ml) (log_10_ SD)	Range
KNUST	Lettuce	5.90 × 10^6^ (±0.65)	2.00 × 10^5^–1.67 × 10^7^
	Manure	3.954 × 10^6^ (±0.74)	2.00 × 10^5^–1.31 × 10^7^
	Soil	6.57 × 10^6^ (±0.69)	3.00 × 10^5^–1.31 × 10^7^
	Irrigation water	3.25 × 10^6^ (±0.57)	3.00 × 10^5^–1.24 × 10^7^

Deduako	Lettuce	1.83 × 10^6^ (±0.49)	2.00 × 10^5^–5.7 × 10^6^
	Manure	3.95 × 10^6^ (±0.71)	2.00 × 10^5^–1.67 × 10^7^
	Soil	4.15 × 10^6^ (±0.75)	2.00 × 10^5^–1.31 × 10^7^
	Irrigation water	4.68 × 10^6^ (±0.71)	3.00 × 10^5^–1.31 × 10^7^

Boadi	Lettuce	5.62 × 10^6^ (±0.56)	5.55 × 10^5^–1.42 × 10^7^
	Manure	2.97 × 10^6^ (±0.51)	2.80 × 10^5^–5.7 × 10^6^
	Soil	4.93 × 10^6^ (±0.60)	4.00 × 10^5^–1.28 × 10^7^
	Irrigation water	4.32 × 10^6^ (±0.69)	2.80 × 10^5^–1.67 × 10^7^

**Table 2:** Mean microbial load of lettuce per week (*n* = 42).

Week	Mean of microbial load (MPN/100 ml)
1	3.18 × 10^5a^
2	4.56 × 10^6e^
3	1.06 × 10^7c^
4	5.23 × 10^6b^
5	1.24 × 10^6d^
6	5.35 × 10^6b^
7	4.10 × 10^6e^

Means in a row with different superscripts are significantly different (*p* < 0.05).

**Table 3:** Antimicrobial sensitivity of *E. coli* isolates (*n* = 225).

Antibiotic	% Resistant	% Susceptible
Ampicillin	91.1	8.1
Tetracyclin	90.6	9.4
Cotrimoxazole	90.2	9.8
Cefuroxime	95.1	4.9
Vancomycin	95.1	4.9
Chloramphenicol	89.4	10.6
Ceftriaxone	94.7	5.3
Cefotaxime	94.2	5.8
Ciprofloxacin	91.5	8.5
Meropenem	94.2	5.8

**Table 4:** Antimicrobial sensitivity of *E. coli* isolates from lettuce (*n* = 60).

Antibiotic	% Resistant	% Susceptible
Ampicillin	90	10
Tetracycline	90	10
Cotrimoxazole	88.3	11.7
Cefuroxime	93.3	6.7
Vancomycin	93.3	6.7
Chloramphenicol	86.6	13.4
Ceftriaxone	95	5
Cefotaxime	91.7	8.3
Ciprofloxacin	90	10
Meropenem	91.7	8.3

**Table 5:** Antimicrobial sensitivity of *E. coli* isolates from manure (*n* = 41).

Antibiotic	% Resistant	% Susceptible
Ampicillin	82.9	17.1
Tetracyline	82.9	17.1
Cotrimoxazole	82.9	17.1
Cefuroxime	90.3	9.7
Vancomycin	87.8	12.2
Chloramphenicol	80.5	19.5
Ceftriaxone	85.4	14.6
Cefuroxime	85.4	14.6
Ciprofloxacin	83	17
Meropenem	85.4	14.6

**Table 6:** Antimicrobial sensitivity of *E. coli* isolates from soil (*n* = 63).

Antibiotic	% Resistant	% Susceptible
Ampicillin	95.3	4.7
Tetracyline	95.2	4.8
Cotrimoxazole	92.1	7.9
Cefuroxime	96.8	3.2
Vancomycin	98.4	1.6
Chloramphenicol	96.9	3.1
Ceftriaxone	96.8	3.2
Cefotaxime	98.4	1.6
Ciprofloxacin	95.2	4.8
Meropenem	98.4	1.6

**Table 7:** Antimicrobial sensitivity of *E. coli* isolates from irrigation water (*n* = 61).

Antibiotic	% Resistant	% Susceptible
Ampicillin	93.5	6.5
Tetracycline	91.8	8.2
Cotrimoxazole	95.1	4.9
Cefuroxime	98.4	1.6
Vancomycin	98.4	1.6
Chloramphenicol	90.1	9.9
Ceftriaxone	98.3	1.7
Cefotaxime	98.4	1.6
Ciprofloxacin	95.1	4.9
Meropenem	98.4	1.6

**Table 8:** Common resistance patterns of *E. coli* (*n* = 225).

Resistance profile	Number of isolates	%
AMPCOTCRXVANCTRCTXCIPMEM	1	0.44
AMPCRXVANCHLCTRCTXCIPMEM	3	1.33
AMPCRXVANCTRCTXCIPMEM	1	0.44
AMPTETCOTCRXCHLCIPMEM	1	0.44
AMPTETCOTCRXCHLCTXCIP	1	0.44
AMPTETCOTCRXVANCHLCTRCIPMEM	2	0.89
AMPTETCOTCRXVANCHLCTRCTXCIP	2	0.89
AMPTETCOTCRXVANCHLCTRCTXCIPMEM	174	77.33
AMPTETCOTCRXVANCHLCTRCTXMEM	4	1.78
AMPTETCOTCRXVANCHLCTXMEM	1	0.44
AMPTETCOTCRXVANCTRCTXCIPMEM	7	3.11
AMPTETCOTCRXVANCTRCTXMEM	2	0.89
AMPTETCOTVANCHLCTRCTXCIPMEM	1	0.44
AMPTETCOTVANCTRCTXCIPMEM	1	0.44
AMPTETCRXVANCHLCTRCTXCIPMEM	2	0.89
COTCRXVANCHLCTRCTXCIPMEM	1	0.44
COTCRXVANCTRCTXCIPMEM	1	0.44
CRXVANCHLCTRCIPMEM	1	0.44
CRXVANCHLCTRCTXCIPMEM	2	0.89
CRXVANCTRCTXCIPMEM	1	0.44
None	9	4

## Data Availability

The data used in the study are available within the manuscript.
